# Hepatocellular carcinoma in a mouse model fed a choline‐deficient, L‐amino acid‐defined, high‐fat diet

**DOI:** 10.1111/iep.12240

**Published:** 2017-09-12

**Authors:** Ayae Ikawa‐Yoshida, Saori Matsuo, Atsuhiko Kato, Yusuke Ohmori, Atsuko Higashida, Eiji Kaneko, Masahiko Matsumoto

**Affiliations:** ^1^ Chugai Research Institute for Medical Science, Inc. Gotemba Japan; ^2^ Fuji‐Gotemba Research Laboratories Chugai Pharmaceutical Co., Ltd. Gotemba Japan

**Keywords:** choline deficiency, fibrosis, hepatocarcinogenesis, non‐alcoholic steatohepatitis, spontaneous liver tumour model

## Abstract

Hepatocellular carcinoma (HCC) is a common cancer worldwide and represents the outcome of the natural history of chronic liver disease. The growing rates of HCC may be partially attributable to increased numbers of people with non‐alcoholic fatty liver disease (NAFLD) and non‐alcoholic steatohepatitis (NASH). However, details of the liver‐specific molecular mechanisms responsible for the NAFLD–NASH–HCC progression remain unclear, and mouse models that can be used to explore the exact factors that influence the progression of NAFLD/NASH to the more chronic stages of liver disease and subsequent HCC are not yet fully established. We have previously reported a choline‐deficient, L‐amino acid‐defined, high‐fat diet (CDAHFD) as a dietary NASH model with rapidly progressive liver fibrosis in mice. The current study in C57BL/6J mice fed CDAHFD provided evidence for the chronic persistence of advanced hepatic fibrosis in NASH and disease progression towards HCC in a period of 36 weeks. When mice fed CDAHFD were switched back to a standard diet, hepatic steatosis was normalized and NAFLD activity score improved, but HCC incidence increased and the phenotype of fibrosis‐associated HCC development was observed. Moreover, when mice continued to be fed CDAHFD for 60 weeks, HCC further developed without severe body weight loss or carcinogenesis in other organs. The autochthonous tumours showed a variety of histological features and architectural patterns including trabecular, pseudoglandular and solid growth. The CDAHFD mouse model might be a useful tool for studying the development of HCC from NAFLD/NASH, and potentially useful for better understanding pathological changes during hepatocarcinogenesis.

## Introduction

Liver cancer is the fifth most common cancer worldwide and the second most common cause of cancer mortality (Torre *et al*. 2015). Hepatocellular carcinoma (HCC) is a major histological subtype and accounts for 70%–85% of primary liver cancers (El‐Serag & Rudolph 2007). The majority of HCC cases are due to chronic viral hepatitis B and C infections. Rigorous efforts to control viral hepatitis are succeeding with the spread of vaccination and innovative drugs (Chang *et al*. 1997; Shiffman *et al*. 2015), and in developed countries, HCC traditionally associated with chronic hepatitis viral infection is expected to decline in the future (Tanaka *et al*. 2008; Yuen *et al*. 2009). However, obesity and metabolic syndrome are increasing in clinical importance in Western countries owing to lifestyle changes (Bugianesi *et al*. 2004), and non‐alcoholic fatty liver disease (NAFLD) and non‐alcoholic steatohepatitis (NASH), which are diseases associated with these lifestyle changes, have emerged as relevant risk factors for HCC (Neuschwander‐Tetri & Caldwell 2003; Gambarin‐Gelwan 2013; Tateishi *et al*. 2015). It has been calculated that between 4% and 22% of HCC cases can be ascribed to NAFLD (Michelotti *et al*. 2013). However, the molecular mechanisms responsible for the NAFLD–NASH–HCC progression are not fully understood.

The greatest risk factor for NAFLD/NASH pathogenesis is fatty liver, which results from an imbalance between lipid deposition and removal driven by the hepatic synthesis of triglycerides and *de novo* lipogenesis (Marchesini *et al*. 2016). Methionine and choline are needed for hepatic secretion of very low‐density lipoproteins (VLDL) (Yao & Vance 1988). Feeding mice a diet deficient in both methionine and choline (MCD diet) induces macrovesicular steatosis, hepatic inflammation and fibrosis, and the MCD model is one of the most common tools for NAFLD/NASH research (Caballero *et al*. 2010). However, that mouse model shows severe body weight loss; therefore, it is difficult to use for long‐term experiments leading up to carcinogenesis (Rizki *et al*. 2006). Another formula with the MCD diet is the choline‐deficient, L‐amino acid‐defined (CDAA) diet. Mice fed the CDAA diet develop steatosis, hepatic inflammation and moderate pericellular fibrosis without body weight loss after 22 weeks (Denda *et al*. 2002). Furthermore, mice fed that diet for 84 weeks develop hepatocellular adenoma (HCA) and HCC. However, in that long‐term model, the development of spontaneous tumours, such as lymphoma and bronchioloalveolar adenoma, is also observed. Therefore, improvement in this diet is crucial for its use in a NAFLD–NASH–HCC progression mouse model.

Recently, Matsumoto, *et al*. have reported that a choline‐deficient, L‐amino acid‐defined, high‐fat diet (CDAHFD) mouse model develops steatosis, steatohepatitis and hepatic fibrosis more rapidly and severely than the conventional models (Matsumoto *et al*. 2013). With this model, the body weight of mice does not decline and shows a gradual increase during 14‐week CDAHFD feeding. Therefore, we thought that it would be possible to use this model to evaluate further development of hepatic change. In terms of exploring the progression from several properties of metabolic syndrome to liver‐specific carcinogenesis, we were interested in examining whether this model can cause HCC. We were also interested in clarifying whether excess fat accumulation in non‐adipose tissues such as the liver leads to the development of HCC in NAFLD and whether HCC represents the outcome of the natural history of chronic liver disease. We examined the effects of long‐term CDAHFD feeding on the development of hepatocarcinogenesis related to NAFLD/NASH in male C57BL/6J mice.

## Materials and methods

### Animals

Specific pathogen‐free male C57BL/6J mice (5 weeks old) were purchased from Japan SLC, Inc. (Shizuoka, Japan) and allowed to acclimatize for 1 week before the start of treatments. At 6 weeks old, the mice were randomly divided into three groups: the SD group (*n* = 40) was fed a commercial standard diet (SD; #CE‐2, CLEA Japan Inc., Shizuoka, Japan); the CDAHFD group (*n* = 50) was fed a choline‐deficient, L‐amino‐acid‐defined, high‐fat diet with 0.1% methionine (CDAHFD; A06071302, Research Diets, New Brunswick, NJ, USA) (Matsumoto *et al*. 2013); and the CDAHFD/SD group (*n* = 6) was fed CDAHFD for 36 weeks, switched back to SD at 37 weeks and fed SD until 48 weeks.

Animals were maintained at 23 ± 3°C with a 12‐h:12‐h light/dark cycle and tap water available *ad libitum*.

### Ethical approval statement

All experimental animal care and handling were performed in accordance with the guidelines of the Institutional Animal Care and Use Committee of Chugai Pharmaceutical Co. Ltd., which is certified by the Association for Assessment and Accreditation of Laboratory Animal Care International. The animal experimental protocol was approved by the Institutional Animal Care and Use Committee at Chugai Pharmaceutical Co. Ltd (Approval No: 14‐011).

### Experimental procedures

All mice were maintained under the above‐mentioned conditions for 12–60 weeks. At each 12‐week sampling point, mice were weighed and then sacrificed by exsanguination under isoflurane anaesthesia. Blood samples were collected from the heart cavities and maintained at −80°C until assayed. The liver was quickly removed and weighed. To assess the extent of macroscopic liver nodules (diameter > 1 mm), we scored each liver on a scale of 0–3 as follows: stage 0, no visible nodules on the liver; stage 1, one to three nodules; stage 2, four to six nodules; and stage 3, seven or more nodules. After that, part of the liver tissue was snap‐frozen on dry ice for hydroxyproline (OH‐Pro) and mRNA analysis, and another small piece of liver was immediately fixed in 10% neutral‐buffered formalin (Wako Pure Chemical Industries, Osaka, Japan) for further histological analysis.

### Biochemical analyses

The plasma levels of alanine:2‐oxoglutarate aminotransferase (ALT), total bilirubin (T‐BIL) and albumin (ALB) were analysed by TBA‐120FR chemistry analyser (Toshiba, Tochigi, Japan). The plasma levels of monocyte chemoattractant protein‐1 (MCP‐1) were measured using a commercial mouse MCP‐1 enzyme‐linked immunosorbent assay (ELISA) Kit (R&D Systems, Minneapolis, MN, USA).

To assess collagen content in the liver, we measured OH‐Pro content by LC/MS/MS. The chopped liver specimens were hydrolysed overnight in 6 N hydrochloric acid at 110°C, diluted with deionized distilled water and filtered. Chromatographic analysis was performed on a Waters Acquity UPLC system (Waters Corp., Milford, MA, USA). Chromatographic separation of trans‐4‐hydroxy‐L‐proline (HP), trans‐4‐hydroxy‐L‐proline‐2,5,5‐d3 (HPd3, surrogate standard) and 3,4‐dehydro‐L‐proline (IS, internal standard) was achieved using an Acquity UPLC BEH Amide column (2.1 mm × 50 mm, 1.7 μm; Waters Corp.) on a Waters Acquity UPLC system. The mobile phase was composed of A (water containing 0.15% formic acid and 10 mM ammonium formate) and B (acetonitrile containing 0.05% formic acid and 2 mM ammonium formate), and a gradient elution was performed as follows: 0–0.15 min, 10% A; 0.15–1.0 min, 10%–22% A; 1.0–2.8 min, 22% A; and 2.8–3.0 min, 22%–10% A. The flow rate was set at 0.4 ml/min. The column temperature and injection volume were set at 40°C and 5 μl respectively. HP, HPd3 and IS were detected by electrospray ionization using multiple‐reaction monitoring in positive mode on a QTRAP 5500 System (AB Sciex, Framingham, MA, USA). The optimal MRM transitions for the precursor ion to the specific product ion [M + H]+ were selected for HP (m/z 132 → 86), HPd3 (m/z 135 → 86) and IS (m/z 114 → 68). analyst 1.5.1 software (AB Sciex) was used to acquire, analyse and process the data. The hepatic OH‐Pro content is expressed in milligrams per gram of tissue (dry weight).

Baseline plasma MCP‐1 or hepatic OH‐Pro content was measured on 22 mice fed SD for 12 or 60 weeks.

### mRNA analyses using quantitative real‐time polymerase chain reaction (qRT‐PCR)

Total RNA was isolated using Isogen reagent (Nippon Gene, Tokyo, Japan), and cDNAs were synthesized from RNAs using a Transcriptor First Strand cDNA Synthesis Kit (Roche Applied Science, Tokyo, Japan) according to the manufacturers’ instructions. Subsequent PCR amplification was performed with an Applied Biosystems StepOnePlus Real‐Time PCR system (Thermo Fisher Scientific Inc., Japan). mRNA expressions of the specific genes were quantified using TaqMan Gene Expression Assays (Thermo Fisher Scientific Inc., Japan) as follows: mouse glyceraldehyde 3‐phosphate dehydrogenase (*Gapdh*; Mm99999915_g1), tumour necrosis factor alpha (*Tnfa*; Mm00443258_m1), transforming growth factor beta 1 (*Tgfb1*; Mm01178820_m1), actin alpha 2 (*Acta2*; Mm00725412_s1; the gene encoding α‐smooth muscle actin), collagen type I (*Col1a1*; Mm00801666_g1), collagen type III (*Col3a1*; Mm01254476_m1), heme oxygenase 1 (*Hmox1*; Mm00516005_m1), alpha fetoprotein (*Afp*; Mm00431715_m1) and glypican 3 (*Gpc3*; Mm00516722_m1). Relative mRNA expression values were calculated using the relative standard curve method normalized to *Gapdh*.

### Hepatic histopathological evaluation

Haematoxylin–eosin (HE) staining and Masson's trichrome (MT) staining were performed on 10% formalin‐fixed and 3‐μm‐thick paraffin‐embedded sections of liver tissue. Right/left medial lobules and areas with macroscopical abnormality in mice fed SD or CDAHFD for 12 weeks and after sampling point, and left lateral lobe in mice fed SD or CDAHFD (including CDAHFD/SD) for 36 weeks and after sampling point were selected as representative sites for histopathological examination of proliferative lesion. Proliferative changes in hepatocytes were diagnosed by three independent pathologists as either regenerative hyperplasia (RH), hepatocellular adenoma (HCA) or hepatocellular carcinoma (HCC), including early hepatocellular carcinoma. For diagnosis of these lesions in this study, the criteria from the International Harmonization of Nomenclature and Diagnosis Criteria (INHAND) project was applied (Thoolen *et al*.^2010^). In addition, using the liver section of 24‐week sampling point of CDAHFD group and 48‐week sampling point of CDAHFD group and CDAHFD/SD group, NAFLD activity score and grading of fibrosis were assessed. HE‐stained liver sections were scored according to the NAFLD activity score (Kleiner *et al*. 2005). MT‐stained sections were used to reveal fibrosis, and hepatic fibrosis was classified into stages 0–3 as follows: stage 0, normal liver sections without fibrosis; stage 1, fibrous expansion of the perisinusoidal or periportal area; stage 2, fibrous expansion of the perisinusoidal and periportal areas; and stage 3, bridging fibrosis.

### Statistical analyses

Data are presented as mean ± standard error of the mean (SEM). Student's *t*‐test, Dunnet's test and Pearson's chi‐squared test were performed using the jmp statistical software package (version 11.2.1, SAS Institute Inc., Cary, NC, USA). Differences were considered significant when *P* values were less than 0.05, 0.01 or 0.001.

## Results

### CDAHFD feeding for 60 weeks resulted in enlarged liver and symptoms of NASH without involving loss of body weight

Six‐week‐old C57BL/6J mice were fed SD or CDAHFD for 12–60 weeks. Initial body weights were 21.6 ± 0.1 g (mean ± SEM; *n* = 90). The body weight of mice fed CDAHFD gradually increased, although at every sampling point, mice fed CDAHFD had lower body weight than mice fed SD (Table 1a). The liver weights in both groups tended to increase with the age of the mice. The ratio of liver weight to body weight was significantly higher in mice fed CDAHFD than in mice fed SD throughout the 60 weeks. Long‐term feeding of CDAHFD induced a continuously enlarged liver without loss of body weight.

**Table 1:** Time course of physiological changes in C57BL/6J mice fed CDAHFD for 60 weeks

Feeding period (weeks)	Body weight (g)		Liver weight (g)		Liver/body weight (%)	
	SD	CDAHFD	SD	CDAHFD	SD	CDAHFD
(a) Body and liver weights						
12	29.3 ± 0.5 (6)	23.6 ± 0.6 (6)***	1.37 ± 0.03 (6)	1.69 ± 0.05 (6)***	4.67 ± 0.07 (6)	7.16 ± 0.12 (6)***
24	34.2 ± 1.3 (6)	24.9 ± 0.6 (6)***	1.66 ± 0.07 (6)	1.75 ± 0.12 (6)	4.86 ± 0.11 (6)	7.06 ± 0.41 (6)***
36	36.3 ± 1.5 (6)	27.2 ± 0.8 (12)***	1.60 ± 0.06 (6)	2.26 ± 0.10 (12)***	4.42 ± 0.10 (6)	8.31 ± 0.25 (12)***
48	40.6 ± 1.2 (6)	29.9 ± 1.1 (11)***	1.94 ± 0.07 (6)	3.01 ± 0.17 (11)***	4.79 ± 0.12 (6)	10.0 ± 0.4 (11)***
60	44.9 ± 1.4 (16)	28.3 ± 0.7 (15)***	2.14 ± 0.14 (16)	3.94 ± 0.27 (15)***	4.71 ± 0.16 (16)	13.8 ± 0.7 (15)***

Feeding period (weeks)	Alanine:2‐oxoglutarate aminotransferase (U/l)		Total bilirubin (mg/dl)		Albumin (g/dl)	
	SD	CDAHFD	SD	CDAHFD	SD	CDAHFD
(b) Biochemical markers						
12	26 ± 8 (6)	273 ± 14 (6)***	0.06 ± 0.00 (6)	0.14 ± 0.01 (6)***	2.78 ± 0.03 (6)	2.83 ± 0.05 (6)
24	28 ± 2 (6)	193 ± 23 (6)***	0.07 ± 0.01 (6)	0.34 ± 0.19 (6)**	2.75 ± 0.10 (6)	2.83 ± 0.06 (6)
36	18 ± 2 (6)	182 ± 21 (12)***	0.09 ± 0.01 (6)	0.29 ± 0.08 (12)***	3.02 ± 0.02 (6)	2.94 ± 0.04 (12)
48	30 ± 3 (6)	277 ± 19 (11)***	0.06 ± 0.01 (6)	0.18 ± 0.01 (11)***	3.30 ± 0.04 (6)	3.31 ± 0.04 (11)
60	39 ± 7 (16)	403 ± 36 (15)***	0.07 ± 0.01 (16)	0.48 ± 0.13 (15)***	3.25 ± 0.06 (16)	3.48 ± 0.08 (15)*

	CDAHFD feeding period (weeks)					
	Baseline	12	24	36	48	60
(c) Plasma MCP‐1 levels and hepatic hydroxyproline contents						
No. of tested mice	22	6	6	12	11	15
MCP‐1 (pg/ml)	6.5 ± 0.8	34.6 ± 2.3***	33.6 ± 4.7***	28.9 ± 2.3***	34.4 ± 2.3***	38.9 ± 1.8***
Hydroxyproline content (μg/mg)	0.49 ± 0.03	2.31 ± 0.30**	2.72 ± 0.32***	3.25 ± 0.30***	4.26 ± 0.31***	4.40 ± 0.53*

Numbers in parentheses indicate No. of tested mice. Values are expressed as means ± SEM.

a: ***P < 0.001 (*vs*. SD, *t*‐test).

b: *P < 0.05, **P < 0.01, ***P < 0.001 (*vs*. SD, *t*‐test).

c: Baseline is the average value of plasma MCP‐1 or hepatic hydroxyproline contents in mice fed SD for 12 weeks or 60 weeks.

c: **P < 0.01, ***P < 0.001 (*vs*. baseline, Dunnet's test).

Table 1b shows serial changes in biochemical markers in plasma. In mice fed CDAHFD, the levels of ALT and T‐BIL increased significantly and remained at high levels for 60 weeks. The plasma ALB levels in mice fed CDAHFD tended to be equal to or higher than those in mice fed SD. Therefore, the livers of mice that were fed CDAHFD for a long period of time could maintain the function of protein synthesis even though there was chronic liver disorder.

The levels of the circulating inflammation marker (plasma MCP‐1) in mice fed CDAHFD persisted at a high concentration (4–6 times baseline) for 60 weeks (Table 1c). The levels of hepatic fibrosis marker (OH‐Pro) in the liver were also significantly higher than baseline level and increased during the period (Table 1c). These data suggested that long‐term CDAHFD feeding could cause chronic systemic inflammation and developing liver fibrosis.

### CDAHFD feeding caused formation of multiple nodules in the liver of each mouse

**Figure 1:**
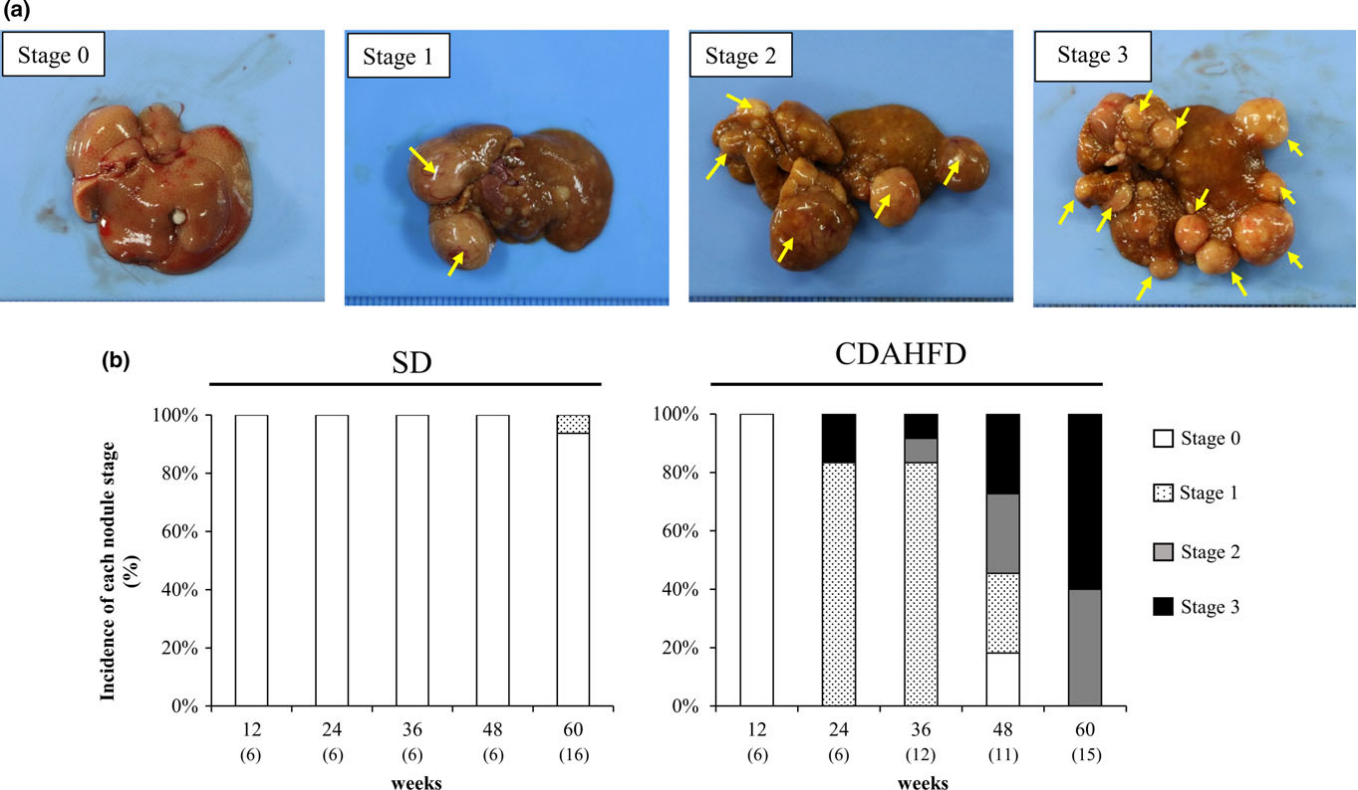
Nodule formation in C57BL/6J mice fed standard diet (SD) or choline‐deficient, L‐amino acid‐defined, high‐fat diet (CDAHFD) for 12, 24, 36, 48 or 60 weeks. (a) Representative macroscopic appearance of liver of each nodule stage. Stage 0, no nodules visible on the liver; stage 1, one to three nodules; stage 2, four to six nodules; and stage 3: seven or more nodules (yellow arrows indicate visible nodules). (b) Incidence of nodule stage by number of weeks on each diet. Numbers in parentheses indicate No. of tested mice. [Colour figure can be viewed at wileyonlinelibrary.com].

The formation of macroscopic nodules in the liver was detected in mice fed CDAHFD. To assess the extent of nodule formation, we assigned each mouse liver a nodule stage of between 0 and 3. Figure 1 shows livers representative of each nodule stage and the incidence of nodule stages by number of weeks on each diet. In mice fed CDAHFD, no nodules were observed in any mice at 12 weeks, and at least one nodule was observed at 24 weeks; after that, the multiplicity of liver nodules increased during 36–60 weeks. The nodules were of various sizes and some nodules were accompanied by necrosis and haemorrhage. The sites at which nodules formed were also different, with the formation of nodules or number of nodules not being biased towards any particular lobe. Among the mice fed SD, on the other hand, only one nodule on the liver was seen in one mouse fed SD for 60 weeks, and no mice except for that one formed any nodules.

### CDAHFD feeding for 24 weeks resulted in NASH histopathological changes and gene expression changes

**Figure 2:**
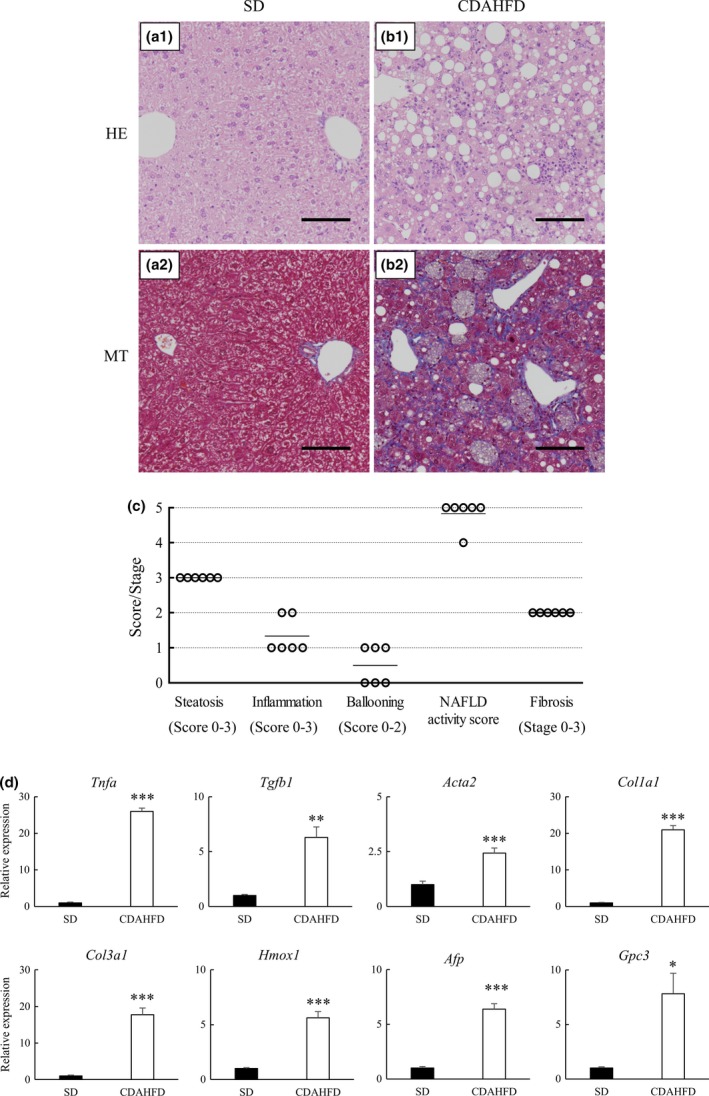
CDAHFD feeding for 24 weeks resulted in NASH histopathological changes and changes in gene expression. Representative liver histopathology in C57BL/6J mice fed standard diet (SD) or choline‐deficient, L‐amino acid‐defined, high‐fat diet (CDAHFD) for 24 weeks. (a‐1 and b‐1) Haematoxylin–eosin (HE) staining; (a‐2 and b‐2) Masson's trichrome (MT) staining. Mice fed CDAHFD showed steatosis, lobular inflammation, hepatocyte ballooning and fibrosis. Scale bars = 100 μm. (c) Histopathological assessment of non‐alcoholic fatty liver in C57BL/6J mice fed CDAHFD for 24 weeks. The morphological features of steatosis (stage 0–3), lobular inflammation (stage 0–3), ballooning (stage 0–2) and fibrosis (stage 0–3) were semiquantitatively evaluated. Subsequently, the NAFLD activity score was applied (<3, non‐NASH; 3–4, possible NASH; and >5, NASH). (d) Assessment of mRNA expression levels of the indicated genes in the nodule‐free areas of liver at 24 weeks by qRT‐PCR normalized to the expression level of *Gapdh* mRNA. Data are means ± SEM (*n* = 6). *P < 0.05, **P < 0.01 and ***P < 0.001 *vs*. SD,* t*‐test. [Colour figure can be viewed at wileyonlinelibrary.com].

We conducted histopathological assessments of NAFLD/NASH at 24 weeks when incidence of nodules had begun to be observed. Figure 2a,b shows representative light micrographs of livers of mice fed SD or CDAHFD at 24 weeks. Severe steatosis, moderate to marked lobular inflammation and hepatocyte ballooning were observed in the livers of mice fed CDAHFD, but not in the livers of mice fed SD. There was no difference in degree of steatosis for each area in the whole hepatic acinus, and distribution of the area of fibrosis was expanded in both the perisinusoidal and periportal areas in the livers of mice fed CDAHFD. Figure 2c shows the NAFLD activity score and fibrosis stage of the liver in each mouse fed CDAHFD at 24 weeks. Based on NAFLD activity score ≥5 together with a global assessment of ‘definite NASH’ (Kleiner *et al*. 2005), five of the six mice fed CDAHFD were diagnosed with NASH. Moreover, all of the mice fed CDAHFD had developed moderate fibrosis in the liver before multiple nodule formation was observed.

The expression of genes associated with inflammation and fibrosis, such as *Tnfa*,* Tgfb1*,* Acta2*,* Col1a1* and *Col3a1*, at 24 weeks was significantly increased in mice fed CDAHFD, compared with mice fed SD (Figure 2d). Furthermore, the expression of *Hmox1* (a gene associated with oxidative stress) and expression of oncofetal markers, such as *Afp* and *Gpc3,* were similarly elevated at 24 weeks in mice fed CDAHFD. These results implied a possible carcinogenic response in the livers of mice fed CDAHFD.

### CDAHFD feeding induced the occurrence of liver tumours after 36 weeks without increasing the incidence of spontaneous tumours in other organs

Table 2 shows the incidence of proliferative change in the liver. Regenerative hyperplasia (RH) began to appear at 24 weeks, and after that, multiple areas of RH were observed in the livers of all mice fed CDAHFD, but not in those fed SD. In mice fed CDAHFD, HCA and HCC began to appear at 36 weeks, and in the weeks after that, 100% of mice had HCA and incidences of HCC were continuously observed until 60 weeks. Moreover, other tumours such as lymphoma and bronchioloalveolar adenoma were not observed in mice fed CDAHFD. On the other hand, in mice fed SD, mice other than the one mouse did not develop HCA or HCC, and bronchioloalveolar adenoma was observed in one other mouse at 60 weeks.

**Table 2:** Microscopic findings in liver of C57BL/6J mice fed CDAHFD for 60 weeks

Feeding period (weeks)	Regenerative hyperplasia		Hepatocellular adenoma		Hepatocellular carcinoma	
	SD	CDAHFD	SD	CDAHFD	SD	CDAHFD
12	0/6^a^	0/6	0/6	0/6	0/6	0/6
24	0/6	2/6	0/6	0/6	0/6	0/6
36	0/6	12/12***	0/6	8/12*	0/6	2/12
48	0/6	11/11***	0/6	11/11***	0/6	1/11
60	0/16	15/15***	0/16	15/15***	1/16	4/15

a Number of mice observed per number of mice used.

*P < 0.05, ***P < 0.001 (*vs*. SD, Pearson's chi‐squared test).

**Figure 3:**
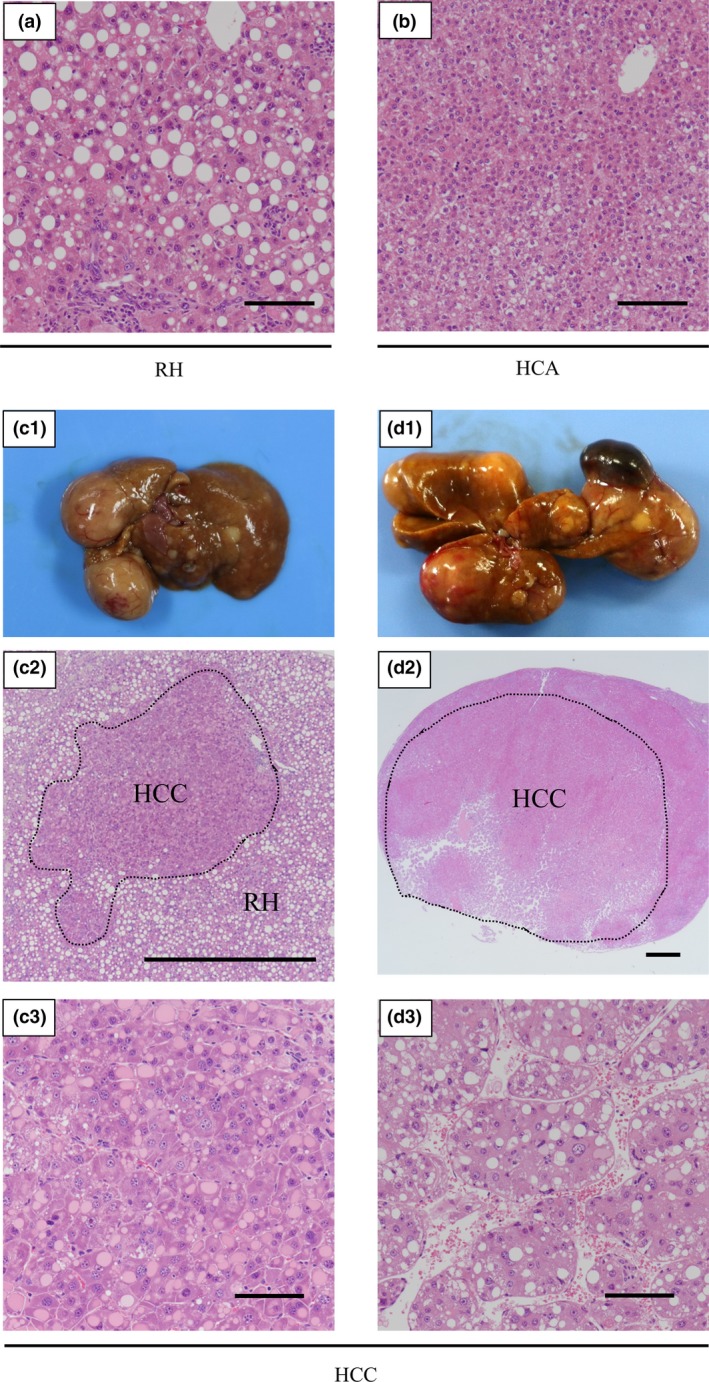
Representative examples of changed liver histopathology (haematoxylin–eosin staining) in mice fed the choline‐deficient, L‐amino acid‐defined, high‐fat diet (CDAHFD) for 60 weeks. (a) Area of regenerative hyperplasia (RH). (b) Area of hepatocellular adenoma (HCA). (c, d) Representative liver that shows hepatocellular carcinoma (HCC) and micrograph of its area of HCC. Scale bars = 100 μm (scale bar in panel c‐2 and d‐2 = 1 mm). [Colour figure can be viewed at wileyonlinelibrary.com].

Figure 3 shows representative examples of altered liver histopathology in mice fed CDAHFD. In areas of RH, steatosis, lobular inflammation and hepatocyte ballooning were observed, but normal lobular architecture was maintained (Figure 3a). Compared to RH areas, areas of HCA showed that steatosis, lobular inflammation and hepatocyte ballooning had disappeared and that normal lobular architecture was broken, with an irregular hepatocyte growth pattern and multiple cell layers (Figure 3b). Moreover, areas of HCC displayed complete disappearance of normal lobular architecture and severe cellular atypia (Figure 3c,d). We observed HCC with a variety of architectural patterns and histological characteristics. The HCC shown in Figure 3c was arising from RH, and the basophilic tumour cells had intracytoplasmic acidophilic inclusion body and shown solid growth. Another HCC shown in Figure 3d was arising without other proliferative changes around. The tumour cells had intracellular vacuole and were organized in large trabeculae (Figure 3d‐2).

### Switching back to standard diet at 37 weeks could improve NASH pathology but could not prevent the occurrence of tumours

**Figure 4:**
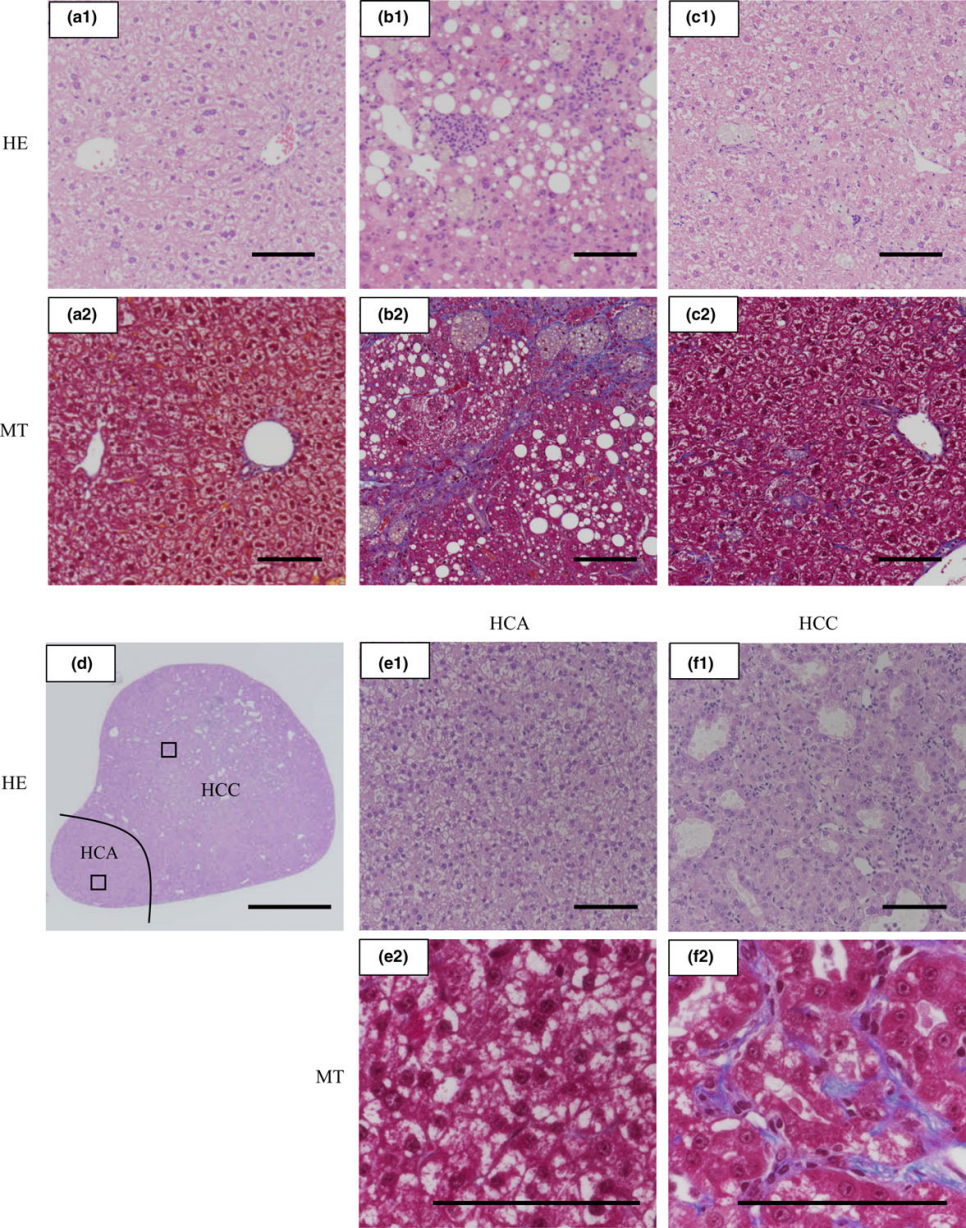
Switching back to standard diet at 37 weeks can improve NASH pathology but cannot prevent the occurrence of tumours. Representative liver histopathology in C57BL/6J mice fed a standard diet (SD) for 48 weeks; a choline‐deficient, L‐amino acid‐defined, high‐ fat diet (CDAHFD) for 48 weeks; or CDAHFD for 36 weeks and then SD until 48 weeks (CDAHFD/SD). (a‐1 and a‐2) Haematoxylin–eosin (HE) staining and Masson's trichrome (MT) staining of SD fed mice. (b‐1 and b‐2) Mice fed CDAHFD for 48 weeks showed steatosis, lobular inflammation, hepatocyte ballooning and bridging fibrosis. (c‐1 and c‐2) Mice switched back to SD showed that steatosis, lobular inflammation and fibrosis were reduced when compared with mice fed CDAHFD for 48 weeks. (d) Micrograph of a nodule in a mouse in the CDAHFD/SD group in which HCA and HCC existed next to each other. (e‐1 and e‐2) HCA region boxed in (d). An irregular hepatocyte growth pattern was observed, but fibrosis was not. (f‐1 and f‐2) HCC region boxed in (d). A pseudoglandular structure was observed and fibrosis penetrates into the gap that is causing structural atypia. Scale bars = 100 μm (scale bar in panel d = 1 mm). [Colour figure can be viewed at wileyonlinelibrary.com].

CDAHFD feeding induced development of HCA and HCC at 36 weeks and beyond, and we evaluated whether this pathology was reversible by setting up a CDAHFD/SD group, which was fed CDAHFD for 36 weeks, switched back to SD at 37 weeks and fed SD until 48 weeks. Body weight, liver weight, NAFLD activity score, fibrosis and liver tumour incidence at 48 weeks in the CDAHFD/SD group are summarized in Table 3. All mice in the CDAHFD/SD group, with the exception of mouse No. 92, rapidly gained body weight after switching back to SD, and the liver‐to‐body weight ratio was tending towards mitigation. The NAFLD activity score of all mice was ≤3, a diagnosis of ‘not NASH’. Fibrosis was restored to mild fibrosis in four of the six mice, but was not improved in the remaining two mice. Figure 4a–c shows representative micrographs of livers of mice in the SD, CDAHFD and CDAHFD/SD groups at 48 weeks. Although moderate hepatocyte ballooning was still observed in the livers of mice switched back to SD, steatosis and lobular inflammation were reduced when compared with mice fed CDAHFD for 48 weeks (Figure 4b‐1,c‐1). In the livers of mice fed CDAHFD for 48 weeks, fibrosis expanded by portal‐to‐portal septa (bridging fibrosis) (Figure 4b‐2). However, in the livers of mouse No. 90 whose fibrosis was improved by switching back to SD, only weak fibrosis was seen (Figure 4c‐2). On the other hand, the tumour development did not improve but rather progressed; almost all mice in the CDAHFD/SD group maintained RH and HCA, and four of six mice developed HCC (Table 3). The HCC was observed not only in liver of the two mice whose fibrosis did not improve (mouse No. 88 and No. 92) but also in liver of the two mice whose fibrosis was improved (mouse No. 90 and No. 93). As shown in Figure 4d–f, micrographs of a nodule in mouse No. 88 show that HCA and HCC were adjacent. In the HCA region, fibrosis was hardly observed, but in the HCC region, fibrosis penetrates into the gap that is causing structural atypia.

**Table 3:** Body and liver weight and liver microscopic findings at 48 weeks in C57BL/6J mice fed CDAHFD for 36 weeks and switched back to SD feeding for 12 weeks after CDAHFD feeding (CDAHFD/SD) and in C57BL/6J mice fed only CDAHFD for 48 weeks (CDAHFD)

	CDAHFD	CDAHFD/SD					
	Mean ± SEM (*n *=* *11)	Mouse ID number					
		No. 88	No. 89	No. 90	No. 91	No. 92	No. 93
Body weight (g)	29.9 ± 1.1	32.2	33.8	34.9	35.4	21.5	34.2
BW gain for 12 weeks (%)	4.1 ± 0.6	+21	+29	+30	+27	–8	+21
Liver weight (g)	3.0 ± 0.2	2.6	3.0	3.2	3.4	1.7	3.3
Liver weight/body weight (%)	10.0 ± 0.4	8.1	8.7	9.1	9.7	7.8	9.6
Histological features							
NAFLD activity score (0–8)	5.5 ± 0.2	1	2	2	3	2	3
Steatosis (0–3)	3.0 ± 0.0	0	0	0	1	0	1
Lobular inflammation (0–3)	1.5 ± 0.2	1	1	1	1	1	1
Ballooning (0–2)	1.0 ± 0.0	0	1	1	1	1	1
Fibrosis (stage 0–3)	2.4 ± 0.2	2	1	1	1	3	1
Incidence of nodular hyperplasia							
Nodular regenerative hyperplasia	100%	+	−	+	+	+	+
Hepatocellular adenoma	100%	+	+	+	+	+	+
Hepatocellular carcinoma	9.1%	+	−	+	−	+	+

## Discussion

In this study, we demonstrated that CDAHFD feeding induced rapid progression towards steatohepatitis with fibrosis, consistent with the results of previous 14‐week studies (Matsumoto *et al*. 2013), and that subsequent feeding up to 60 weeks developed an ascending process of liver tumorigenesis in C57BL/6J male mice. In this model, liver cancer consistently developed after intermediate steps that mimicked the natural history of NAFLD/NASH in humans. We suggest that this CDAHFD mouse model may be a useful tool for studying the development of HCC from steatohepatitis without the need for any genetic modification or xenobiotic compounds.

Although there are several reports of mouse models that develop NAFLD–NASH–HCC progression, many of those were in genetically modified mice (Watanabe *et al*. 2007; Nakanishi *et al*. 2008; Itoh *et al*. 2011). Genetically modified mice are useful tools for evaluating relationships between the target gene and the pathology; however, there may be several potential impacts of the gene modification, and the pathophysiological processes in HCC progression cannot be clarified. NAFLD–NASH–HCC mouse models involving administration of xenobiotic compounds show rapid progression of NASH to HCC, and the HCC incidence is relatively high in each (Fujii *et al*. 2013; De Minicis *et al*. 2014). Unfortunately, because these chemical substances themselves are genotoxic, there is a possibility that the cancer is caused by these substances. Therefore, these models cannot be used to elucidate mechanisms of carcinogenesis by physiological progress from NASH.

On the other hand, nutritional challenges, such as a high‐fat diet (HFD) (VanSaun *et al*. 2009; Tajima *et al*. 2013), CDAA diet (Denda *et al*. 2002; De Minicis *et al*. 2014) or a high‐fat, high‐fructose diet (Murine ALIOS Model) (Dowman *et al*. 2014), can induce NASH‐associated tumour development in wild‐type mice. However, in the HFD mouse model, HFD feeding induces slowly developing NASH and weak fibrosis over 60 weeks, and develops only up to HCA with no HCC, in 80 weeks. Mice fed CDAA develop NASH pathology at 12 weeks and moderate fibrosis; however, it takes 84 weeks until HCC develops, and the development of tumours of other organs is increased. Therefore, the development of HCC could involve influences of carcinogenesis not only from NASH progression with fibrosis but also from age‐related systemic changes. In the Murine ALIOS Model, although HCC can be observed at 48 weeks, progression of steatosis and lobular inflammation is weak and 48 weeks is needed before the mice can be diagnosed with NASH, which is the same period as carcinogenesis. Furthermore, even after 48 weeks, there are some individuals in which fibrosis and NASH do not occur at all. Therefore, the carcinogenesis is not necessarily induced by NAFLD/NASH pathological progression.

In the CDAHFD mouse model, the onset of NASH and fibrosis is very early: NASH pathology develops at 3 weeks and fibrosis is first observed at 6 weeks; the onset of these is very early (Matsumoto *et al*. 2013). Hepatocyte ballooning and severe fibrosis were observed and these histological changes were similar to those seen in human NASH (Matteoni *et al*. 1999; Angulo *et al*. 1999). This pathological progression continued without any individual difference and involved no loss of body weight, and HCC developed at 36 weeks, faster than in other diet‐induced models. Moreover, HCC incidence was continuously observed from 36 weeks until 60 weeks in mice fed CDAHFD without carcinogenesis in any other organ. C57BL/6J mice are known to be a strain resistant to spontaneous liver tumours (Bursch *et al*. 2004); therefore, we expect that CDAHFD feeding will be able to induce carcinogenesis in other strains and transgenic mice according to various experimental purposes.

To examine the mechanisms underlying the progression of NASH pathology and the proliferative changes in hepatocytes, we evaluated histopathological changes and changes in gene expression in C57BL/6J mice fed CDAHFD for 24 weeks. It was previously reported that in this CDAHFD model, there is rapid progression towards steatohepatitis with fibrosis in the short period of 6 weeks, with continued liver damage and development of fibrosis (Matsumoto *et al*. 2013). Also, emergence of RH and increased levels of oncofetal marker (*Afp* and *Gpc3*) were confirmed at 24 weeks. The *Afp* and *Gpc3* are known as a marker of hepatic progenitor/oval cells (Abelev & Eraiser 1999; Grozdanov *et al*. 2006). Then, it was suggested that regenerative cell proliferation accompanied with these precursor cells occurred in response to continuing hepatocellular damage. Regenerative cell proliferation can contribute to liver carcinogenesis caused by a choline‐deficient diet (Abanobi *et al*. 1982; Giambarresi *et al*. 1982; Chandar & Lombardi 1988). Moreover, oxidative stress accumulating in the DNA following HFD and CDAA feeding is reported to induce hepatocellular carcinogenesis (Nakae *et al*. 1990; Denda *et al*. 2002; Matsuzawa‐Nagata *et al*. 2008). In our mouse model, increased levels of oxidative stress marker (*Hmox1*) were also confirmed at 24 weeks. We postulate that the preparation for carcinogenesis was well advanced at this point, leading to tumour development at 36 weeks.

To further confirm this observation, we switched mice from CDAHFD back to SD at 37 weeks, when the tumour development had been confirmed, and evaluated the relationship between the NASH histopathological changes and tumour development. As shown in HFD‐induced NASH rodent models (Nakamura *et al*. 2012), the pathological features of NASH such as steatosis and inflammation were immediately reversed in all mice that had previously been fed CDAHFD, but fibrosis could not be reversed in all of the mice at that point (Table 3). As for tumour development, switching back to SD was not able to completely suppress the incidence of HCA and HCC. With the CDAA‐induced NASH–HCC rat model, it was reported that tumour development could be prevented by switching the diet back to SD at 24 weeks (Nakae *et al*. 1992). However, in that rat model, no HCC emergence was observed at the time point of 24 weeks. On the other hand, in our CDAHFD mouse model, at 36 weeks, when the diet switched back to SD, the emergence of HCA or HCC was confirmed in some mice. Therefore, some early HCC could already exist at 36 weeks and this time was considered as a point of no return in cancer development.

HCC incidence in mice in the CDAHFD/SD group was significantly higher than that in mice fed CDAHFD for 48 weeks. It would be considered permissible to speculate on the subject of the metabolic environment that promotes HCC cell growth in SD feeding. During CDAHFD feeding, a low level of methionine and a choline deficiency was reported to induce a remarkable accumulation of hepatic triglyceride (Matsumoto *et al*. 2013), and mRNA expression of endogenous fatty acid synthesis has been downregulated as a compensatory response (data not shown). Cancer cells are commonly known to increase *de novo* fatty acid synthesis, in a manner functionally related to the glycolytic pathway (Kuhajda 2000), and enhanced fatty acid synthesis is reported to be involved in cancer proliferation (Menendez & Lupu 2007). Thus, after the diet was switched back to SD in the CDAHFD/SD group, we speculate that fatty acid synthesis in the liver recovered and the liver became a more growth friendly environment for neoplastic cells than was the liver in mice fed only CDAHFD.

Microscopically, there was a nodule in a mouse in the CDAHFD/SD group in which HCA and HCC were adjacent, and then fibrosis was observed in the HCC region but not in the HCA region. Although it is well known that the accumulation of collagen produced by myofibroblasts is primarily responsible for liver fibrosis as a consequence of chronic hepatitis (Bataller & Brenner 2005), some myofibroblasts have recently been reported to imply HCC progression (van Zijl *et al*. 2009; Okabe *et al*. 2016). Mazzocca *et al*. indicated that HCC cells promote stromal fibroblast transdifferentiation into myofibroblasts and that the myofibroblasts contribute to HCC's proliferative, migratory and invasive properties (Mazzocca *et al*. 2011). Probably because a mutual relationship already existed between the HCC cells and myofibroblasts in this mouse, switching from CDAHFD back to SD might not have been able to prevent development of their fibrosis‐associated HCC phenotype. On the other hand, the two mice with HCCs in the CDAHFD/SD group showed weak fibrosis (fibrosis stage 1). Despite the well‐documented association between steatohepatitis or cirrhotic fatty liver disease and HCC (Bugianesi *et al*. 2002; Bruix & Sherman 2005; Hashimoto *et al*. 2009), recent evidence from clinical studies has suggested an association between non‐cirrhotic fatty liver and HCC (Kawada *et al*. 2009; Starley *et al*. 2010; Ikura *et al*. 2011). Our CDAHFD mouse model might be a useful tool for studying the relationship between fatty liver disease and HCC in both cirrhotic and non‐cirrhotic livers.

In summary, we have proposed a mouse model that shows a NAFLD–NASH–HCC progression similar to the natural course of human pathology. In addition, we have provided information of the interesting time point at which the NASH pathology can recover immediately, but tumour development is irreversible. This model is applicable to various mouse strains; therefore, use of this model could greatly contribute to elucidation of tumour development caused not only by NAFLD/NASH but also by chronic liver disease.

## Conflict of Interests and Funding Statement

The authors of this work are employees of Chugai Pharmaceutical Co., Ltd. or Chugai Research Institute for Medical Science Inc., and have not received financial support from any other institution. The authors declare no conflict of interest.
